# Glycemic variation in uncontrolled Graves’ disease patients with normal glucose metabolism: Assessment by continuous glucose monitoring

**DOI:** 10.1007/s12020-018-1820-0

**Published:** 2018-12-04

**Authors:** Gu Gao, Feng-fei Li, Yun Hu, Reng-na Yan, Bing-li Liu, Xiao-mei Liu, Xiao-fei Su, Jian-hua Ma, Gang Hu

**Affiliations:** 1 Department of Endocrinology, Nanjing First Hospital, Nanjing Medical University, Nanjing, China; 2 Jiangsu Key Laboratory of Neurodegeneration, Department of Pharmacology, Nanjing Medical University, Nanjing, China

**Keywords:** Graves’ disease, Glycemic variation, Continuous glucose monitoring, Mean amplitude of glycemic excursions

## Abstract

**Purpose:**

To observe the glycemic variation (GV) in uncontrolled Graves’ disease (GD) patients with normal glucose metabolism measured by continuous glucose monitoring (CGM).

**Methods:**

This was a single-center, open-label, observational study. From January 2017 to October 2017, 20 GD patients with normal glucose metabolism and 24 healthy control subjects were recruited. Serum samples were obtained at 0, 30, and 120 min after oral glucose loading for glucose, insulin, and C-peptide level measurements. Fasting plasma fasting free triiodothyronine (FT3), free thyroxin (FT4), and thyroid stimulating hormone concentrations were also detected. All participants were subjected to a 3-day CGM after baseline data were collected. The primary endpoint was the difference in the mean amplitude of the glycemic excursions between the two groups.

**Results:**

Compared with the healthy subjects, the GD patients had higher mean amplitude of glycemic excursions (MAGE) (*P* < 0.01). Multiple linear stepwise regression analysis showed that FT4 level was an independent factor for the MAGE. Interestingly, the GD patients had a significant prolongation in the time to peak glucose, especially after breakfast (*P* < 0.01), and the elevation in the incremental area under the curve of glucose after breakfast till 4 hours later.

**Conclusions:**

Uncontrolled GD patients with normal glucose metabolism had a greater GV, and the FT4 level may contributed to the increased GV.

## Introduction

Impaired glucose tolerance, insulin resistance, and increased insulin secretion were found in patients with hyperthyroidism [1–5]. Known factors, responsible for the abnormal glucose tolerance, including aberrant metabolic rate, increased endogenous gluconeogenesis, excessive insulin secretion, enhanced glucose absorption, and dawn phenomenon, which are affected by the thyroid hormones [6–8].

Using the oral glucose tolerance test (OGTT) criteria screening newly diagnosed Graves’ disease (GD) patients, the incidence of pre-diabetes and diabetes were 41.3% and 11.3%, respectively, which were higher than those of using glycosylated hemoglobin (HbA_1c_) criteria [9]. Continuous glucose monitoring (CGM) provides 288 glucose signals throughout a period of 24 hrs. Mean amplitude of glycemic excursions (MAGE), and standard deviation (SD) obtained from CGM were used to assess the daily glycemic variations (GVs) [10, 11]. Studies demonstrated that GD patients with type 2 diabetes (T2D) had increased fasting and postprandial blood glucose monitored by CGM, and the glucose profile was improved along with the normalization of thyroid function [12], which indicated that the thyroid function might be a risk factor of GV. However, the GV in GD patients with a normal glucose metabolism was not well clarified perhaps because of scarcity of studies using CGM. We then performed a single-center, open, and observational study. In this study, we compared the 24 hrs GVs using CGM between GD patients with normal glucose tolerance (NGT) and healthy control subjects.

## Materials and methods

The study was performed in the Department of Endocrinology, Nanjing First Hospital, Nanjing Medical University, from January 2017 to October 2017. The research protocol had passed the approval of the hospital ethics committee on 09 September 2016. Informed consent was obtained from all individual participants included in the study. A total of 33 GD patients and 44 age-gender matched healthy control subjects were enrolled.

Uncontrolled GD patients with NGT were enrolled. Patients were excluded if they had a history of diabetes mellitus or abnormal glucose tolerance, or they had severe complications such as hyperthyroid heart disease, hyperthyroidism crisis, or they had severely impaired liver and kidney function, anemia and psychiatric disorders, or they were pregnant or planning to become pregnant, or they had received systemic hormone therapy within 3 months, or those with acute infection or stress in the previous 4 weeks, or they had abuse of alcohol or drugs.

All the recruited GD patients were admitted as inpatients. OGTT using 75 g of glucose (dissolved in 200 ml water) was performed at baseline. Serum samples were obtained at 0, 30, and 120 min after oral glucose loading for blood glucose, insulin, C-peptide level measurements. C-peptide, insulin, and glucose concentrations were measured centrally at the central laboratory in Nanjing First Hospital, Nanjing Medical University. Fasting free triiodothyronine (FT3), free thyroxin (FT4), and thyroid stimulating hormone (TSH) were detected by chemiluminescence (Abbott Laboratories). The normal value of FT4, FT3, and TSH used in our hospital were 9.0–19.0 pmol/L, 2.63–5.7 pmol/L, and TSH 0.35–4.94 IU/mL, respectively. HbA_1c_ was measured by a DiaSTAT HbA_1c_ analyzer (Bio-Rad, Hercules, CA).

After OGTT was performed, all the participants in both groups were subjected to a 3-day retrospective CGM (Sof-sensor, CGMS-Gold, Medtronic Incorporated, Northridge, USA) in the hospital by the specialist nurse. All the subjects were instructed to maintain usual physical activity and received meals consisting of a total daily caloric intake of 25 kcal/kg/day (60% carbohydrate, 20–25% lipid, and 15–20% protein, respectively), and all the subjects were instructed to have breakfast, lunch and dinner at 0700, 1100, and 1700, respectively, during the 3 days of CGM period. The CGM was calibrated four times per day by the finger-stick blood glucose concentrations.

The primary endpoint was the difference in MAGE between the two groups. The MAGE was calculated manually for each patient by measuring the arithmetic mean of the ascending and descending excursions between consecutive peaks and nadirs for the same 24-hrs period, and only absolute excursion values > 1SD were considered as described previously [13, 14]. The differences in terms of 24-hrs mean blood glucose (MBG), SD, coefficient of variation (CV%), the incremental area under curve (AUC) and the time spent in glucose > 7.8, > 10.0, and < 3.9 mmol/L, the hourly MBG, homeostasis model assessment for insulin resistance (HOMA-IR) (fasting plasma glucose (FPG) × fasting insulin/22.5), and homeostasis model assessment for beta cell function (HOMA-β) (FINS×20/(FPG−3.5)) between the two groups were also recorded. Hypoglycemia was defined as glucose concentration < 3.9 mmol/L monitored by CGM. The detailed study protocol was described in the study flow chart (Table 1).

**Table 1:**
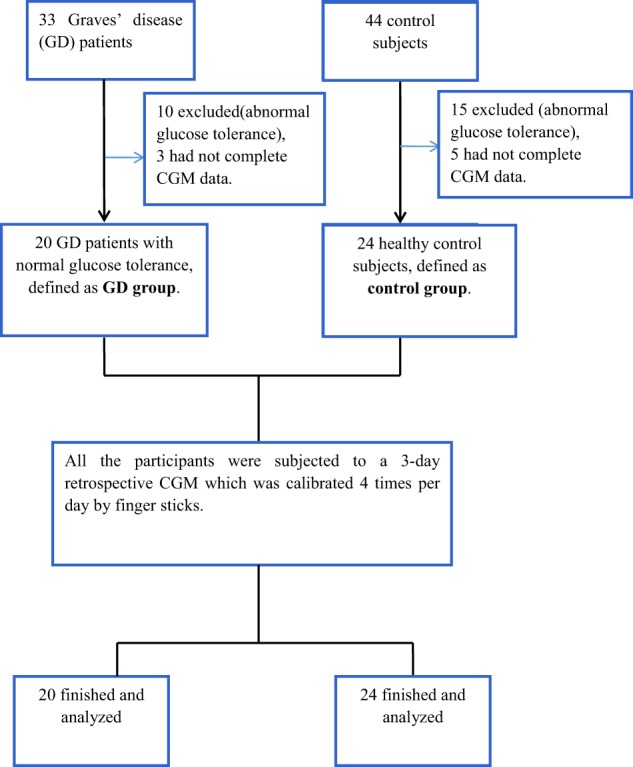
Study flow chart

The study was registered with ClinicalTrials.gov identifier: ChiCTR-ROC-17010362.

### Statistical methods

Data were analyzed with the SPSS PASW Statistics 22 Package. Shapiro–Wilk test was used to assess the distribution of data. Normally distributed and continuous variables were presented as mean ± SD. Non-normal distributed variables were presented as interquartile range). The Mann–Whitney *U* test was used to compare non-normal distribution of data. An independent samples *t* test was used to compare the differences between the two groups. General linear model for repeated measurements was used in the comparison of groups. Bonferroni correction was followed. We used correlation coefficients and multiple linear regression analyses to examine the interrelationships among the GVs and thyroid hormones. All comparisons were two-sided at the 5% significance level. *P* value < 0.05 was considered to be statistically significant.

## Results

### Baseline characteristics

Between January 2017 and October 2017, a total of 33 GD patients and 44 healthy control subjects were recruited for the study. Thirty-three patients were excluded from this study: 13 were from GD group and 20 from control group. They were either owing to abnormal glucose tolerance, or had not completed the CGM data. Thus, there were 20 GD patients with NGT (8 men and 12 women) in the GD group, and 24 healthy control subjects (10 men and 14 women) in the control group (Table 2).

**Table 2:** Baseline for the study subjects in the two groups

Items	GD group	Control group	*P*
*N*	20	24	/
Sex	Male 8, female 12	Male 10, female 14	0.87
Age (year)	39.8 ± 13.6	40.5 ± 12.7	0.84
BMI (kg/m^2^)	20.7 ± 3.0	21.7 ± 5.5	0.46
HbA_1c_ (%)	5.2 ± 0.3	5.2 ± 0.3	0.60
TSH (mIU/L)	0.01 ± 0.00	1.68 ± 0.74	0.00
FT3 (pmol/L)	26.53 ± 12.51	4.54 ± 0.46	0.00
FT4 (pmol/L)	45.43 ± 14.09	13.49 ± 1.21	0.00
HOMA-IR	2.1 ± 1.1	1.9 ± 0.9	0.54
HOMA-β	84.1 ± 44.4	75.6 ± 34.1	0.47

*N* number, *BMI* body mass index, *HbA*_*1c*_ glycosylated hemoglobin, *TSH* thyroid-stimulating hormone, *FT3* free triiodothyronine, FT4 free thyroxine, HOMA-IR homeostasis model assessment for insulin resistance, HOMA-β homeostasis model assessment for beta-cell function

**Fig. 1:**
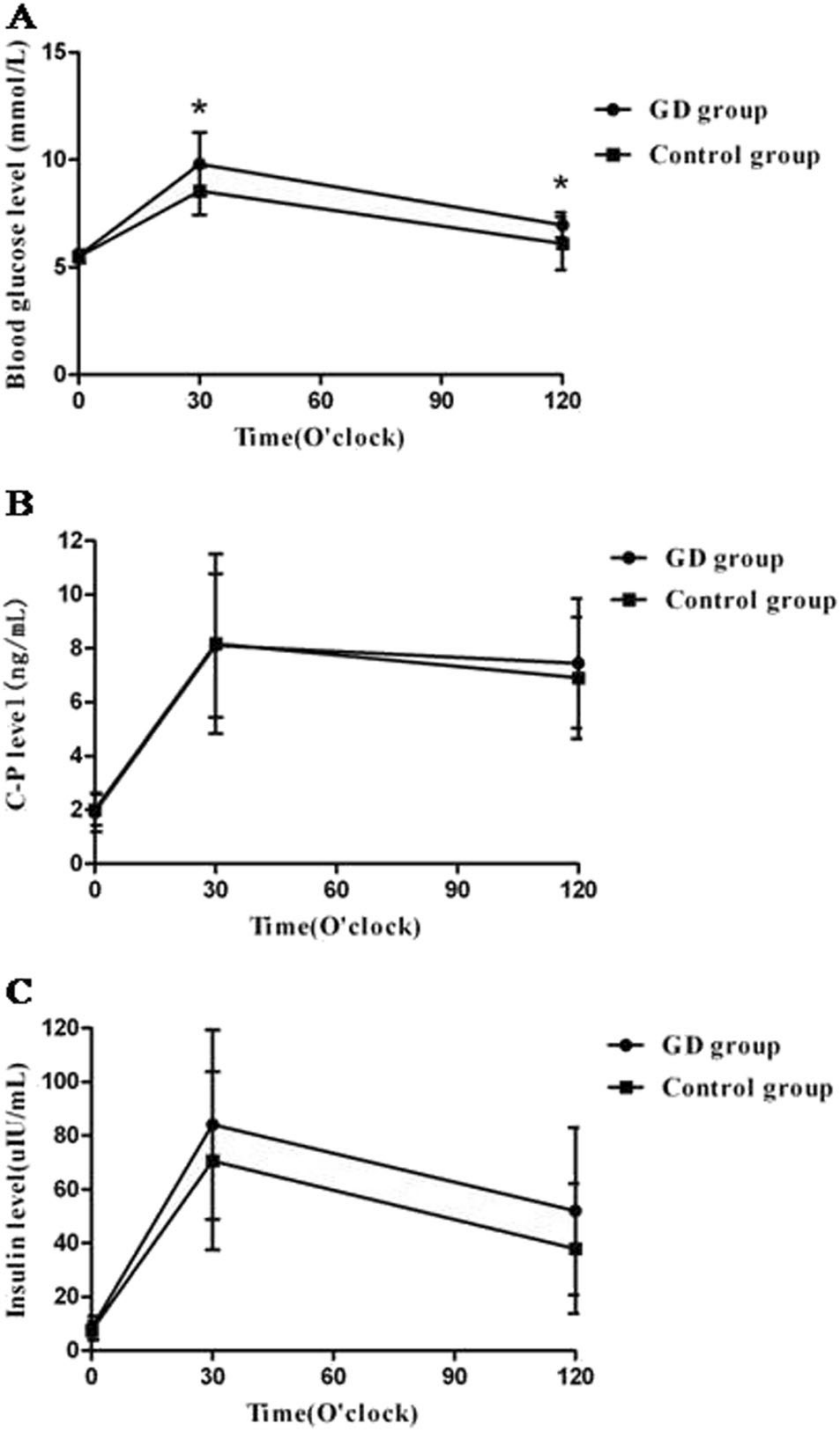
The blood glucose **a**, C-peptide **b**, and insulin levels **c** at 0, 30, and 120 min after glucose loading in the two groups

There were no significant demographic differences in age, sex, body mass index and HbA_1c_ between the two groups at baseline (Table 2). OGTT data showed that the blood glucose levels of 30 and 120 min after glucose loading in GD patients were statistically significantly higher than those in the healthy control subjects (9.8 ± 1.5 vs. 8.6 ± 1.1 mmol/L, 7.0 ± 0.6 vs. 6.1 ± 1.2 mmol/L, *P* < 0.01, respectively). Moreover, the FPG levels between the two groups were similar (5.5 ± 0.3 vs. 5.5 ± 0.3 mmol/L, *P* > 0.05) (Fig. 1a). In addition, there were no significant differences either in fasting and postprandial C-peptide or insulin concentrations (Fig. 1b, c), or in HOMA-IR and HOMA-β between the two groups (*P* > 0.05, respectively) (Table 2).

### GV profiles

**Fig. 2:**
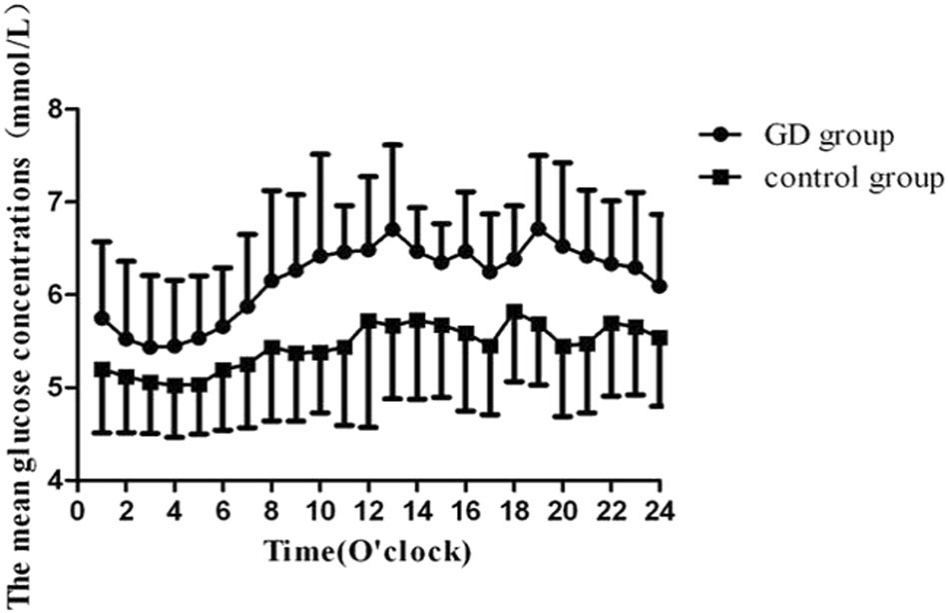
The average glucose concentrations per hour in the study subjects

CGM data showed that the 24-hrs MAGE, MBG, SD, and CV% were significantly increased in GD group compared with those in the control group (2.0 ± 1.2 vs. 1.2 ± 0.5 mmol/L, 6.2 ± 0.5 vs. 5.4 ± 0.6 mmol/L, 0.8 ± 0.4 vs. 0.5 ± 0.2 mmol/L, *P* < 0.01, respectively, and 0.1 ± 0.1 vs. 0.1 ± 0.0, *P* < 0.05). In addition, there were significant differences in the hourly MBG between the two groups (all *P* < 0.05) with exception of 0200 and 0300 (Fig. 2). Although GD patients had higher peak glucose concentrations after three meals (breakfast: 7.6 ± 1.3 vs. 6.1 ± 0.9 mmol/L, *P* < 0.01, lunch: 7.9 ± 1.2 vs. 6.3 ± 1.0 mmol/L, *P* < 0.01, dinner: 7.5 ± 1.0 vs. 6.3 ± 1.0 mmol/L, *P* < 0.01). They exhibited significant prolongation in the time to peak glucose after breakfast and dinner (128.0 ± 60.8 vs. 81.0 ± 16.9 min, 115.0 ± 43.0 vs. 80.6 ± 23.3 min, *P* < 0.01, respectively) compared with healthy control subjects. Of significant importance, we observed that the subjects in GD group had dramatically increased glucose levels from 0400.

In this study, all the subjects got up around 0600, had breakfast, lunch at 0700 and 1100, respectively. We also analyzed the GV profiles from 0700 to 1100 between the two groups. Although there were no differences in the incremental AUC of 2 hrs postprandial after three meals between the two groups, GD patients showed gradually elevated the incremental AUC of glucose after breakfast till 4 h (1hrs: 18.8 ± 8.8 vs. 2hrs: 49.3 ± 18.1 vs. 3 hrs: 88.1 ± 21.7 vs. 4 hrs: 139.0 ± 29.0 mmol/L/day, *P* < 0.05, respectively).

The incremental AUC above 7.8 mmol/L (0.0 (0.0, 0.0) vs. 0.0 (0.0, 0.0)), 10.0 mmol/L (0.0 (0.0, 0.0) vs. 0.0 (0.0, 0.0)) and below 3.9 mmol/L (0.0 (0.0, 0.0) vs. 0.0 (0.0, 0.0)) were similar in subjects between the two groups (*P* > 0.05, respectively). In addition, the GD patients spent significant more time in glucose concentrations above 7.8 mmol/L than those of healthy control subjects (25.0 (0.0, 125.0) min vs. 0.0 (0.0, 0.0) min, *P* < 0.01). We did not observe any difference in time spent in glucose concentrations above 10.0 mmol/L (0.0 (0.0, 0.0) min vs. 0.0 (0.0, 0.0) min, *P* > 0.05) or below 3.9 mmol/L (0.0 (0.0, 0.0) min vs. 0.0 (0.0, 0.0) min, *P* > 0.05) in subjects between the two groups.

### Correlation analysis

We analyzed the correlation of the FT3 and the FT4 levels with GVs and other parameters. On logistic analysis, plasma FT3 level was positively correlated with MBG, SD, MAGE, CV%, Glu 30, and INS 120 (*r* = 0.53, *P* < 0.01; *r* = 0.54, *P* < 0.01; *r* = 0.46, *P* < 0.01; *r* = 0.43, *P* < 0.01; *r* = 0.36, *P* < 0.05; *r* = 0.35, *P* < 0.05, respectively). Plasma FT4 level was positively correlated with MBG, SD, MAGE, CV%, Glu 30, and Glu 120 (*r* = 0.49, *P* < 0.01; *r* = 0.49, *P* < 0.01; *r* = 0.51, *P* < 0.01; *r* = 0.39, *P* < 0.01; *r* = 0.40, *P* < 0.01; *r* = 0.32, *P* < 0.05, respectively), and negatively correlated with BMI (*r* = −0.36, *P* < 0.05).

We have performed the statistical correlation of thyroid hormones with HOMA-IR and HOMA-β. However, we did not observe either plasma FT3 or FT4 level correlated with HOMA-IR (*r* = 0.25, *P* > 0.05; *r* = 0.02, *P* > 0.05, respectively) and HOMA-β (*r* = 0.24, *P* > 0.05; *r* = −0.00, *P* > 0.05, respectively).

Multiple linear stepwise regression analysis showed that plasma FT4 level was an independent factor for MAGE (Beta = 0.50, *P* < 0.01).

## Discussion

In this study, we found that GD patients with NGT had an increase in GVs in terms of MAGE and SD compared with those of healthy control subjects.

Patients with hyperthyroidism had a much higher rate of developing abnormal glucose tolerance and diabetes mellitus than the general population using OGTT or HbA_1c_ diagnostic criteria [9, 15, 16]. In this study, we used OGTT to evaluate abnormal glucose tolerance in the GD patients instead of HbA_1c_, which may avoid the underestimated prevalence of diabetes in the GD patients caused by the alteration of erythrocytes in circulation [17, 18].

Using the CGM data, the GD patients with T2D exhibited not only elevated postprandial hyperglycemia, but also fasting hyperglycemia which might be caused by the dawn phenomenon [12]. Moreover, studies indicated that thyroid function may be a risk factor for glycemic control in GD patients [12, 19]. However, the knowledge of GV in GD patients with NGT is limited.

In this study, CGM data showed that GD patients with normal glucose metabolism had increased GVs in terms of MAGE and SD, which was significantly higher than those of healthy control subjects. Of significant importance, we observed that the subjects in GD group had dramatically increased glucose levels from 0400, prolongation in the time to peak glucose, and higher peak glucose concentrations after breakfast. Our findings agreed with the previous study which indicated a significant increase in glucose concentration after 0400 in GD patient with T2D [12]. The mal-rhythm of glucose metabolism in GD patients may be caused by the increased insulin-antagonizing hormone such as growth hormone, insulin resistance, and gluconeogenesis [20, 21]. However, we did not observe any difference in HOMA-IR or HOMA-β between the two groups, which might be the reason that the sample size was relative modest.

Studies indicated that GD patients had excessive insulin secretion in response to increased gluconeogenesis or insulin-antagonizing hormones [6, 22]. The higher insulin concentrations might contribute to hypoglycemia in GD patients with or without diabetes [23–25]. However, we did not observe any differences in the incremental AUC below 3.9 mmol/L between the two groups. Future studies are needed to identify whether hypoglycemia is a common symptom in the GD patients.

FT3 and FT4 were identified as risk factors for higher plasma glucose levels [26]. Interestingly, our data indicated that the FT4 levels were positively correlated with MAGE. Although our data are not conclusive, it does give some indication that the thyroid hormone may influence the GVs.

Our study still has other limitations. First, the study only observed the Chinese population, so the situation might not be the same for other populations. Second, we did not observe for a long time period to determine whether GV could be improved after therapy. Third, we had no data clarifying the mal-rhythm of glucose metabolism in GD patients.

In conclusion, GD patients with normal glucose metabolism had greater glycemic fluctuations when compared with those of healthy control subjects. Our data also indicated that the FT4 level may contribute to the increased GV.
